# Expandable Cages for Lumbar Interbody Fusion: A Narrative Review

**DOI:** 10.3390/jcm13102889

**Published:** 2024-05-14

**Authors:** Soo-Bin Lee, Jonghun Yoon, Sung-Jun Park, Dong-Sik Chae

**Affiliations:** 1 Department of Orthopedic Surgery, Catholic Kwandong University International St. Mary’s Hospital, Incheon 22711, Republic of Korea; sumanzzz@ish.ac.kr; 2 Department of Mechanical Engineering, Hanyang University, Ansan 15588, Republic of Korea; 3 School of Mechanical, Automotive and Aeronautical Engineering, Korea National University of Transportation, Chungju 27469, Republic of Korea

**Keywords:** expandable cages, lumbar interbody fusion, degenerative spinal diseases

## Abstract

Lumbar fusion surgery for treating degenerative spinal diseases has undergone significant advancements in recent years. In addition to posterior instrumentation, anterior interbody fusion techniques have been developed along with various cages for interbody fusion. Recently, expandable cages capable of altering height, lordotic angle, and footprint within the disc space have garnered significant attention. In this manuscript, we review the current status, clinical outcomes, and future prospects of expandable cages for lumbar interbody fusion based on the existing literature. Expandable cages are suitable for minimally invasive spinal surgeries. Small-sized cages can be inserted and subsequently expanded to a larger size within the disc space. While expandable cages generally demonstrate superior clinical outcomes compared to static cages, some studies have suggested comparable or even poorer outcomes with expandable cages than static cages. Careful interpretation through additional long-term follow-ups is required to assess the utility of expandable cages. If these shortcomings are addressed and the advantages are further developed, expandable cages could become suitable surgical instruments for minimally invasive spinal surgeries.

## 1. Introduction

Lower back pain ranks among the foremost causes of disability worldwide, and its predominant etiology is lumbar degenerative spinal disease [1,2], which encompasses various pathological changes in the lumbar spine, including disc degeneration, facet joint arthritis, segmental slip, and spinal stenosis. Lumbar degenerative spinal diseases such as lumbar spondylolisthesis and spinal stenosis significantly affect the quality of life of several patients [3,4,5,6,7]. As the global elderly population continues to grow, there is an increasing number of individuals affected by lumbar degenerative spinal diseases, leading to an increased demand for treatment aimed at enhancing their quality of life [8,9]. In general, conservative treatments such as medication, injections, and physical therapy are implemented in the early stages of the disease, and these interventions are effective in patients with mild symptoms. If the conservative measures do not result in any improvement, surgery may be required [10,11]. Surgical treatment options can be broadly categorized into decompression alone and decompression with instrumented fusion. When spinal instability is confirmed, and extensive decompression is required, instrumented fusion procedures may be necessary [12,13].

Lumbar spinal fusion surgery traditionally involves posterolateral fusion [14], which induces fusion between the facet joints and transverse processes. However, lumbar interbody fusion procedures have been developed to ensure definite fusion between vertebral bodies. The first posterior lumbar interbody fusion (PLIF) was performed in 1944 by Briggs and Milligan using autologous small chip bones derived from spinous processes and the ilium [15]. However, PLIF remained unpopular until the 1970s owing to technical difficulty and surgical complications. Since Steffee reported in 1988 that the addition of pedicle screw fixation led to increased stability and higher fusion rates, PLIF has been widely performed in clinical practice [16].

Lumbar interbody fusion is a surgical procedure aimed at achieving bony fusion between the superior and inferior vertebral bodies by removing the intervertebral disc and inserting a spacer or cage containing bone or bone fusion material into the disc space [17]. This technique can be approached from the anterior or posterior direction. Various bone graft materials such as demineralized bone matrix, allograft bone, and bone morphogenetic protein-2 can be used in conjunction with cages [18].

Minimally invasive surgical techniques have recently been widely adopted for spinal surgeries. Traditionally, interbody fusion procedures such as PLIF involved a posterior approach with extensive facet joint resection, followed by insertion of a cage after traction of the nerve roots and dura mater. Subsequently, transforaminal lumbar interbody fusion (TLIF), which provides access through the space between the nerve roots and dura mater via a posterior approach, was developed and is widely used [19]. Furthermore, minimally invasive approaches from the anterior and lateral corridors have gained popularity, including procedures such as oblique lumbar interbody fusion (OLIF) and direct lateral interbody fusion (DLIF), which are frequently performed [17,20]. These advancements in surgical techniques are aimed at minimizing tissue disruption and reducing the overall invasiveness of spinal procedures [21,22].

Recently, expandable lumbar interbody cages have been developed to facilitate minimally invasive surgeries. The advantage of expandable lumbar interbody cages lies in their ability to increase the intervertebral disc space height and lordotic angle in comparison to traditional static cages. This review provides a detailed examination of the current status, limitations, and potential advancements of expandable lumbar interbody cages in the context of minimally invasive spinal surgeries.

## 2. Methods

The PubMed database was searched from 2016 to 2024 to identify and review recent articles on expandable cages for lumbar interbody fusion. The following combination of keywords was used: “expandable” AND “cage” AND “lumbar” AND “interbody” AND “fusion”. Of 117 search results, 53 results including not relevant articles, case reports, commentaries were excluded. In total, 64 articles (5 meta-analyses, 6 basic studies, 7 review articles, and 46 clinical studies) that discussed expandable lumbar interbody cages were identified. Of these, we selected and reviewed 18 representative and well-researched papers. Additionally, we manually explored the cited studies’ references. Our search was confined to journals published in English.

## 3. History of Lumbar Interbody Cages

In 1943, Briggs first attempted posterior interbody fusion of the lumbar spine using autogenous bone [15,23]. In the mid-1970s, Bagby attempted anterior interbody fusion of the cervical spine to treat horses with “Wobbler syndrome” (cervical myelopathy) by filling a cylindrical metal container called the “Bagby basket” with autogenous bone and reported an 88% fusion rate [24,25,26,27]. Following this, Kuslich adapted this method by creating a titanium cage, which was first used as a standalone cage in interbody fusion surgery for patients with lumbar disc disease under the name “BAK cage” (Bagby and Kuslich) in 1989 and reported a fusion rate of 91% [28].

Subsequently, titanium cages became widely used in interbody fusion surgeries of the lumbar spine. However, they produced significant artifacts on magnetic resonance scans, making it difficult to evaluate and monitor nerve roots and soft tissues [29]. In 1987, Williams et al. developed a poly-ether ether ketone (PEEK) polymer, which became popular owing to its ability to address the limitations of titanium cages [30]. PEEK cages were deemed sufficiently strong, offered a balance of strength without excessive rigidity, and demonstrated durability against wear and fatigue. Moreover, they exhibited natural radiolucency, making them compatible with standard imaging techniques. In essence, PEEK possessed all the essential attributes to serve as a substitute for titanium implants [31]. In 1999, Brantigan introduced the first PEEK implants reinforced with carbon fibers, known as the Brantigan I/F cage [32,33]. Since then, an array of PEEK implants have been introduced, and cage technology has significantly improved in the last decades.

Compared with titanium cages, PEEK cages have the advantage of lower elastic modulus, which reduces the risk of stress shielding, and radiolucency. However, PEEK is hydrophobic, which means that it does not fully fuse with bone. To address these limitations, three-dimensional (3D) printed titanium cages with porous structures have been developed recently [34]. In addition, expandable interbody cages have been developed to increase the lordotic angle and intervertebral disc height of the fusion segments [35].

## 4. Current Status of Expandable Cages

**Figure 1:**
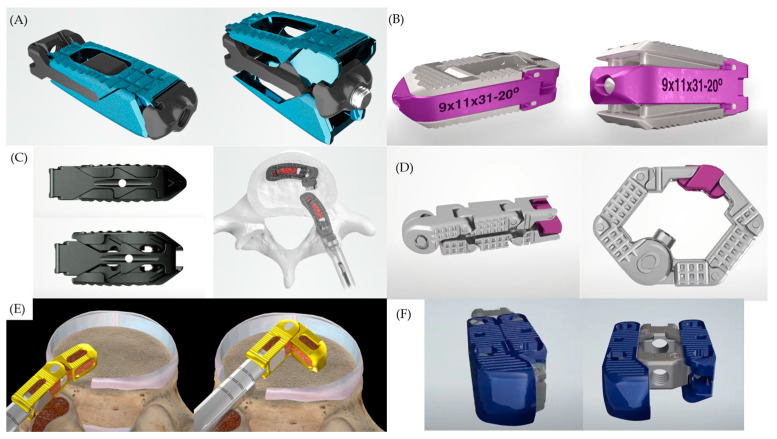
Images of various expandable cages currently available on the market. (**A**) The SABLE (Globus Medical Inc., Audubon, PA, USA) in the fully collapsed and expanded state. (**B**) The TLX (NuVasive Inc., San Diego, CA, USA) in the fully collapsed and expanded state. (**C**) The Altera (Globus Medical Inc.) in the fully collapsed and expanded state, and with articulation, it is positioned horizontally at the anterior aspect of the endplate. (**D**) The MLX (NuVasive Inc.) in the fully collapsed and expanded state with a hexagonal shape. (**E**) The FLXFit15 (CoreLink Surgical, St. Louis, MS, USA) articulates into L-shape within the disc space. (**F**) The Dual-X (Amplify Surgical Inc., Irvine, CA, USA) expands in dual directions, increasing both height and footprint.

In recent years, the use of expandable cages in lumbar interbody fusion has been gradually increasing. Expandable cages can be used in anterior or lateral approaches such as anterior lumbar interbody fusion (ALIF), OLIF, and DLIF or in posterior approaches such as PLIF and TLIF [35]. Historically, the posterior approach is a familiar corridor for spine surgeons and is still the most commonly used approach. Consequently, expandable cages inserted through the posterior approach are the most widely utilized and are actively being developed. Various types of expandable cages are manufactured by different companies. However, the different types of expandable cages generally operate on the basis of similar principles, focusing on altering the lordotic angle, height, and footprint within the disc space. Early versions of expandable cages predominantly featured a simple design with a limited number of components and used internal screws to expand the anterior part of the cage [36]. However, this simplistic structure posed challenges in achieving significant changes in the lordotic angle, height, and footprint. Recently, more complex expandable cages with locking systems have been developed, which allow greater expansion than that of early versions of expandable cages [37]. Expandable cages with a low height and narrow width are inserted into the disc space, which expand in both height and width within the disc space [38,39]. Some products also allow for adjustment of the lordotic angle, typically by increasing the height of the anterior portion of the cage more than that of the posterior portion to increase the lordotic angle [40]. Bone fusion material can be injected into the cage after insertion into the disc space and expansion can be achieved inside the disc space using a special instrument tailored to fit the cage [41]. The following section describes a few of the representative examples of the currently used expandable cages. Images of these representative examples are shown in Figure 1.

The SABLE (Globus Medical Inc.) is an expandable cage developed for PLIF or TLIF surgeries that features endplate contact surfaces made of 3D printed titanium. This feature aims to promote robust bone fusion between the implant and vertebral body. This expandable cage can be inserted at a minimum height of 6 mm and continuously increased to 14 mm. Additionally, it can increase the lordosis angle by up to 22° [42]. The TLX (NuVasive Inc.) is another expandable cage designed for TLIF. The starting height of the implant ranges from 7–14 mm, and this cage can increase the maximum lordosis angle by up to 20° [43]. The Altera (Globus Medical Inc.) is an articulating expandable TLIF cage, which when positioned horizontally at the anterior aspect of the endplate can increase in height by up to 4 mm [44].

Some cages use the TLIF approach and can expand their footprint using the medial-lateral (M-L) expansion method. The MLX (NuVasive Inc.) can expand in a hexagonal shape and achieve a footprint similar than that of an ALIF cage. In a biomechanical cadaver study, an MLX inserted through the TLIF approach demonstrated stability comparable to that of the static PEEK ALIF cage [45]. While this cage allows for M-L expansion, it does not alter the height or lordosis angle. The FLXFit15 (CoreLink Surgical) is a cage that articulates into an L-shape. Through its articulating structure, it can expand the footprint area and achieve an effect similar to that of two TLIF cages. It can increase the anterior height by 4 mm and create a lordosis angle of up to 15° [46,47]. The Dual-X (Amplify Surgical Inc.) is a cage capable of both lateral and vertical expansions. It was designed with lordotic angles ranging from 0°–15°; however, the angle is not adjustable. Its advantages include the creation of a wide footprint and large-center bone graft chamber. Cages for TLIF, PLIF, and lateral lumbar interbody fusion procedures are available [39,48].

## 5. Advantages of Expandable Cages

Expandable cages theoretically offer several advantages. A larger cage area with ample bone graft material is widely recognized to increase fusion rates and reduce the risk of cage subsidence. While posterior approaches offer the advantage of nerve decompression and instrumentation along with cage insertion, accessing the disc space can be challenging due to neural and bony structures, which limits the use of large cages. Inserting a small cage into the disc space and then expanding it to occupy a larger area within the disc space allows for more stable anterior support and ample bone graft material, which theoretically leads to increased fusion rates and reduced subsidence risk [35,49,50,51,52].

Height expansion is also a significant advantage. In posterior approaches, such as PLIF or TLIF, narrow corridors make it difficult to use large cages. Attempting to use oversized cages in tight spaces can cause nerve damage due to excessive traction. Inserting a small cage safely into the disc space and then expanding its height allows for more efficient surgery. The use of taller cages in interbody fusion is crucial for foraminal decompression and restoration of foraminal height, particularly for addressing foraminal stenosis [53]. Additionally, the insertion of taller cages increases the tension in the annulus, promoting more stable anterior bone fusion and indirect decompression of the spinal canal [41,54,55].

**Figure 2:**
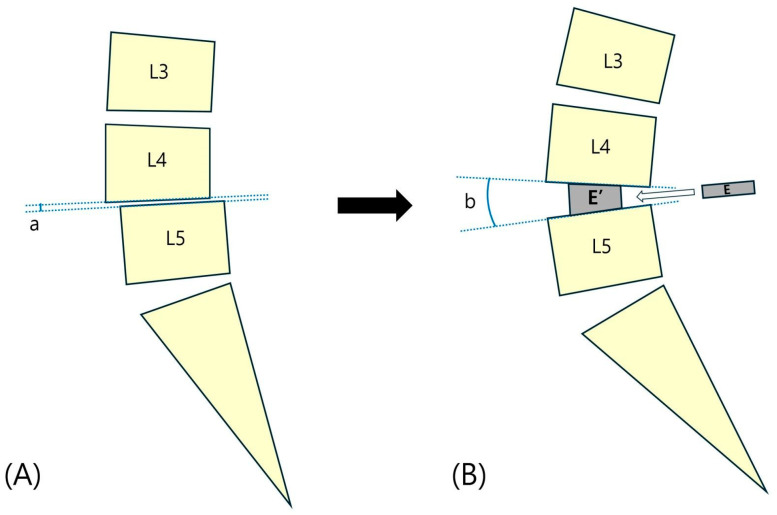
A schematic drawing of the restoration of segmental lordosis and height. (**A**) Typical degenerative lumbar spine with L4-5 spondylolisthesis and disc space narrowing. Loss of lordosis in the L4-5 segmental angle (a) is noted. (**B**) L4-5 segmental angle (b) and height are restored to their normal values by inserting and expanding an expandable cage. Restoration of L4-5 segmental lordosis affects the improvement of overall sagittal balance. The expandable cage can be safely inserted in a collapsed state through a narrow disc space and can be expanded to create a sufficient segmental lordotic angle. a, Pre-operative L4-5 segmental angle. b, Post-operative L4-5 segmental angle. E, Expandable cage in a collapse state. E’, Expandable cage in an expanded state.

Improving sagittal alignment through restoration of lordosis is essential [56]. The lordosis angle of the lumbar spine is a critical parameter. Early spine surgeries, such as those using Harrington rods, often neglect sagittal alignment considerations, resulting in flat back syndrome, which is characterized by flattened fusion sites and subsequent chronic back pain [57]. To restore lumbar lordosis during spinal fusion, it is advisable to use cages with a significant lordotic angle; however, posterior approaches face limitations due to narrow corridors. Expandable cages can be safely inserted in a collapsed state and expanded to create lordotic angles of >20° [37]. Therefore, even without the use of anterior or lateral approaches, achieving substantial lordotic angles is possible using expandable cages [37,39]. A schematic drawing depicting the restoration of segmental lordosis and height is presented in Figure 2.

## 6. Clinical Outcomes of Expandable Cages

Recent studies have reported favorable clinical outcomes in patients who underwent lumbar interbody fusion using expandable cages. Weinstein et al. [47] reported the outcomes of 37 patients who underwent single- or two-level open TLIF surgery using an expandable cage (FLXFit15; CoreLink Surgical) over a 2-year follow-up period. No device-related complications or subsidence were observed. In addition, the average disc height increased by 49% or 6.1 mm. Hawasli et al. [58] indicated that minimally invasive transforaminal lumbar interbody fusion (MIS-TLIF) with an expandable cage resulted in more significant and sustained restoration of the disc height, foraminal height, and index-level segmental lordosis than that with MIS-TLIF with a static cage, particularly in patients with collapsed disc space. Vaishnav et al. [59] reported better outcomes with expandable cages compared to static cages in immediate postoperative disc height restoration and lordosis maintenance in a study of 171 patients undergoing MIS-TLIF surgery. Park and Heo [39] reported the preliminary results of 10 patients who underwent biportal endoscopic TLIF surgery using a dual-direction expandable titanium cage that increased the height and footprint. No subsidence or collapse was noted up to 6 months postoperatively. Kucharzyk et al. [40] compared static and expandable cages in 100 patients and reported that the expandable cage group showed greater radiographic differences in disc height, foraminal height, and lordosis. Superior patient-reported outcomes such as Oswestry Disability Index and Visual Analog Scale scores were reported at the 2-year follow-up compared to those for static cages. Nonunion (6% for expandable cage vs. 12% for static cage) and revision rates (4% for expandable cage vs. 8% for static cage) were were lower in the expandable cage group than that in the static cage group, although the difference was not statistically significant.

Contrary to the aforementioned reports, some studies also suggest that expandable cages may have drawbacks compared to static cages or raise doubts about the cost-effectiveness of expandable cages. Stickley et al. [60] investigated 252 patients who underwent single-level TLIF surgery and reported a significant difference in intraoperative subsidence between the static (11.8%) and expandable (29%) cage groups. Moreover, there was no statistically significant difference between the two groups in terms of overall lumbar lordosis, fusion rate, or postoperative subsidence rate. In terms of fusion rate, both the expandable (94%) cage group and static (92.7%) cage groups showed a high fusion rate of over 90%. Yee et al. [61] conducted a study on 89 patients who underwent TLIF surgery and reported no difference between expandable and static cages in terms of segmental and lumbar lordosis. According to a systematic review and meta-analysis conducted by Alvi et al. [62], no significant difference was observed in the clinical and radiological parameters, including fusion rate, subsidence rate, and change in lumbar lordosis, between patients undergoing MIS-TLIF with expandable cages and those with static cages; it was also mentioned that the higher costs associated with expandable cages may not necessarily justify the use of expandable cages.

The clinical outcomes associated with expandable cages have thus far demonstrated mixed results. Regarding the clinical results of lumbar interbody fusion surgery, a multitude of factors, including surgeon proficiency, surgical technique, and the individual patient’s physical condition, may exert influence, beyond the type of cage. Future well-controlled studies that consider these variables will provide a clearer understanding of the effectiveness of expandable cages.

## 7. Complications and Drawbacks of Expandable Cages

Despite their various advantages, expandable cages are associated with several complications. Stein et al. [63] reported a case of failure with a PEEK-expandable cage (StaXx; Spine Wave Inc., Shelton, CT, USA). This cage allows for height adjustment by inserting a low-profile cage and filling it with a 1 mm wafer. Although the position of the cage was appropriate immediately after surgery, computed tomography (CT) performed 1 year later revealed posterior retropulsion of the wafer, which led to subsequent reoperation. Kim et al. [64] also documented a similar wafer retropulsion issue with the same expandable cage. The use of wafer-type expandable cages is currently uncommon. However, if the cage causes mechanical issues within the body, as was observed in the aforementioned cases, revision surgery may be necessary. Therefore, caution should be exercised when manipulating expandable cages within the body.

Subsidence is also a significant issue associated with expandable cages. Chang et al. [65] reported a significantly higher incidence of subsidence with expandable cages, at 19.7%, compared to 5.4% with static cages in the long-term follow-up of patients who underwent TLIF surgery. Additionally, among the 148 cases in which expandable cages were used, collapse over time was observed in four cases. Stickley et al. [61] also noted higher intraoperative subsidence with expandable cages than that with static cages in patients who underwent TLIF surgery. Given the conflicting results regarding the subsidence of expandable cages, a cautious approach is warranted. Excessive expansion of the cages within a narrow disc space may damage the vertebral body endplates, especially in patients with osteoporosis or osteopenia [66], underscoring the need for careful consideration during the expansion process.

Difficulty in facilitating bone grafting has also been cited as an issue with expandable cages. Unlike static cages, where the bone fusion material is pre-filled, expandable cages are inserted into the body and expanded before bone fusion material is inserted using a device, making dense packing challenging. Additionally, concerns have been raised about the potential of expandable cages to cause stress shielding, and their high cost has been highlighted as a significant issue [50].

## 8. Future of Expandable Cages

The widespread adoption of expandable cages in the future remains uncertain. However, similar to other surgeries, as lumbar interbody fusion increasingly leans towards minimally invasive techniques, the use of expandable cages suitable for minimally invasive surgery may gradually increase in the future. The advantage of inserting expanding cages with low heights and narrow dimensions to occupy an ideal height and area within the disc space is evident. As with all surgical specialties, spinal surgery is anticipated to ultimately transition towards minimally invasive techniques with advancements in surgical methods. TLIF surgeries are performed using minimally invasive approaches, often without the need for traditional incisions, but rather through endoscopic minimal incisions. Expandable cages are believed to clearly demonstrate their advantages in minimally invasive spinal surgeries. Expandable cages must enhance these advantages and address their drawbacks.

Currently, most expandable cages are made of metals due to their mechanical properties requiring transformation. However, metals such as titanium can cause artifacts on magnetic resonance imaging and CT, hindering the accurate assessment of the surrounding tissues such as nerves. The adoption of PEEK material in cages has addressed this issue. Although it may not be feasible to replace all components responsible for the movement of expandable cages with radiolucent PEEK material, several clinical advantages could be achieved if some parts, such as the endplate, were made of PEEK material.

Additionally, research on 3D-printed surface modifications to further promote bone fusion and investigation of bioactive substances is necessary. In addition to vertebral body fusion through bone fusion material in the empty space of the cage, if the cage itself were to directly fuse with the bone tissue of the vertebral body and the disc space at the surface, more stable and reliable bone fusion can be expected. High rates of bone fusion have been reported for 3D-printed static titanium cages, with new results emerging annually [67,68]. Through 3D surface treatment of expandable cages, even higher rates of bone fusion are anticipated to be achieved compared to those with previous methods.

Further studies are required on structures that increase the height, lordosis angle, and footprint to optimal levels. Development of an expandable cage with a larger footprint, height, and lordotic angle than those currently available could lead to more effective surgeries and improved clinical outcomes for patients. Integration with state-of-the-art technologies, such as artificial intelligence, augmented reality, and virtual reality, which are under extensive research [69,70], would be helpful for developing patient-specific expandable cages. Finally, considering the potential limitation of patient access due to high costs, product pricing will be a significant factor in the future of expandable cages.

## 9. Conclusions

With advancements in spinal device manufacturing technology, expandable cages for lumbar interbody fusion have been developed and used widely in recent years. Theoretical advantages of using expandable cages include increasing the height and lordosis angle of the disc space and widening the endplate footprint area to enable optimal interbody fusion. While many clinical outcomes support the superiority of expandable cages over static cages, some reports also suggest that the differences may not be significant or that the risk of issues such as subsidence could be higher with expandable cages. Therefore, whether expandable cages will truly be “game changers” in lumbar interbody fusion remains unclear. If certain drawbacks of expandable cages are further addressed in the future, they are anticipated to be more widely used in interbody fusion surgeries and that patients may experience more satisfactory clinical outcomes following surgery.

## Data Availability

No new data were created or analyzed in this study. Data sharing is not applicable to this article.

## References

[1] GBD 2017 Disease and Injury Incidence and Prevalence Collaborators (2018). Global, Regional, and National Incidence, Prevalence, and Years Lived with Disability for 354 Diseases and Injuries for 195 Countries and Territories, 1990–2017: A Systematic Analysis for the Global Burden of Disease Study 2017. Lancet.

[2] Hoy D., March L., Brooks P., Woolf A., Blyth F., Vos T., Buchbinder R. (2010). Measuring the Global Burden of Low Back Pain. Best Pract. Res. Clin. Rheumatol..

[3] Knezevic N.N., Candido K.D., Vlaeyen J.W.S., Van Zundert J., Cohen S.P. (2021). Low Back Pain. Lancet.

[4] Katz J.N., Zimmerman Z.E., Mass H., Makhni M.C. (2022). Diagnosis and Management of Lumbar Spinal Stenosis: A Review. JAMA.

[5] Lurie J., Tomkins-Lane C. (2016). Management of Lumbar Spinal Stenosis. BMJ.

[6] SenGupta D.K., Herkowitz H.N. (2005). Degenerative Spondylolisthesis: Review of Current Trends and Controversies. Spine.

[7] Park J., Ahn D.K., Choi D.J. (2024). Treatment Concept and Technical Considerations of Biportal Endoscopic Spine Surgery for Lumbar Spinal Stenosis. Asian Spine J..

[8] Lim Y.Z., Chou L., Au R.T.M., Seneviwickrama K.M.D., Cicuttini F.M., Briggs A.M., Sullivan K., Urquhart D.M., Wluka A.E. (2019). People with Low Back Pain Want Clear, Consistent and Personalised Information on Prognosis, Treatment Options and Self-Management Strategies: A Systematic Review. J. Physiother..

[9] Ge L., Pereira M.J., Yap C.W., Heng B.H. (2022). Chronic Low Back Pain and Its Impact on Physical Function, Mental Health, and Health-Related Quality of Life: A Cross-Sectional Study in Singapore. Sci. Rep..

[10] Atlas S.J., Delitto A. (2006). Spinal stenosis: Surgical versus nonsurgical treatment. Clin. Orthop. Relat. Res..

[11] Pearson A.M., Lurie J.D., Tosteson T.D., Zhao W., Abdu W.A., Weinstein J.N. (2013). Who Should Undergo Surgery for Degenerative Spondylolisthesis? Treatment Effect Predictors in Sport. Spine.

[12] Kwon J.W., Moon S.H., Park S.Y., Park S.J., Park S.R., Suk K.S., Kim H.S., Lee B.H. (2022). Lumbar Spinal Stenosis: Review Update 2022. Asian Spine J..

[13] Gupta M.C., Bridwell K.H. (2020). Textbook of Spinal Surgery.

[14] Tajima N., Chosa E., Watanabe S. (2004). Posterolateral Lumbar Fusion. J. Orthop. Sci..

[15] Briggs H., Milligan P.R. (1944). Chip Fusion of the Low Back Following Exploration of the Spinal Canal. J. Bone Joint Surg..

[16] Steffee A.D., Sitkowski D.J. (1988). Posterior Lumbar Interbody Fusion and Plates. Clin. Orthop. Relat. Res..

[17] Mobbs R.J., Phan K., Malham G., Seex K., Rao P.J. (2015). Lumbar Interbody Fusion: Techniques, Indications and Comparison of Interbody Fusion Options Including Plif, Tlif, mi-Tlif, olif/atp, Llif and Alif. J. Spine Surg..

[18] Chang S.Y., Kang D.H., Cho S.K. (2023). Innovative Developments in Lumbar Interbody Cage Materials and Design: A Comprehensive Narrative Review. Asian Spine J..

[19] Kim Y.H., Ha K.Y., Kim Y.S., Kim K.W., Rhyu K.W., Park J.B., Shin J.H., Kim Y.Y., Lee J.S., Park H.Y. (2022). Lumbar Interbody Fusion and Osteobiologics for Lumbar Fusion. Asian Spine J..

[20] Lee W.M., You K.H., Kang M.S., Kim J.H., Park H.J. (2023). Oblique Lumbar Interbody Fusion with Selective Biportal Endoscopic Posterior Decompression for Multilevel Lumbar Degenerative Diseases. Asian Spine J..

[21] Kim H., Chang B.S., Chang S.Y. (2022). Pearls and Pitfalls of Oblique Lateral Interbody Fusion: A Comprehensive Narrative Review. Neurospine.

[22] Kim Y.H., Ha K.Y., Rhyu K.W., Park H.Y., Cho C.H., Kim H.C., Lee H.J., Kim S.I. (2020). Lumbar Interbody Fusion: Techniques, Pearls and Pitfalls. Asian Spine J..

[23] Jaslow I.A. (1946). Intercorporal Bone Graft in Spinal Fusion After Disc Removal. Surg. Gynecol. Obstet..

[24] Bagby G.W. (1988). Arthrodesis by the Distraction-Compression Method Using a Stainless Steel Implant. Orthopedics.

[25] DeBowes R.M., Grant B.D., Bagby G.W., Gallina A.M., Sande R.D., Ratzlaff M.H. (1984). Cervical Vertebral Interbody Fusion in the Horse: A Comparative Study of Bovine Xenografts and Autografts Supported by Stainless Steel Baskets. Am. J. Vet. Res..

[26] Otero Vich J.M. (1985). Anterior Cervical Interbody Fusion with Threaded Cylindrical Bone. J. Neurosurg..

[27] Crawley G.R., Grant B.D., White K.K., Barbee D.D., Gallina A.M., Ratzlaff M.H. (1988). A Modified cloward’s Technique for Arthrodesis of the Normal Metacarpophalangeal Joint in the Horse. Vet. Surg..

[28] Kuslich S.D., Ulstrom C.L., Griffith S.L., Ahern J.W., Dowdle J.D. (1998). The Bagby and Kuslich Method of Lumbar Interbody Fusion. History, Techniques, and 2-Year Follow-Up Results of a United States Prospective, Multicenter Trial. Spine.

[29] McGilvray K.C., Waldorff E.I., Easley J., Seim H.B., Zhang N., Linovitz R.J., Ryaby J.T., Puttlitz C.M. (2017). Evaluation of a Polyetheretherketone (Peek) Titanium Composite Interbody Spacer in an Ovine Lumbar Interbody Fusion Model: Biomechanical, Microcomputed Tomographic, and Histologic Analyses. Spine J..

[30] Williams D.F., McNamara A., Turner R.M. (1987). Potential of Polyetheretherketone (Peek) and Carbon-Fibre-Reinforced Peek in Medical Applications. J. Mater. Sci. Lett..

[31] de Kunder S.L., Rijkers K., Caelers I.J.M.H., de Bie R.A., Koehler P.J., van Santbrink H. (2018). Lumbar Interbody Fusion: A Historical Overview and a Future Perspective. Spine.

[32] Brantigan J.W., Steffee A.D., Lewis M.L., Quinn L.M., Persenaire J.M. (2000). Lumbar Interbody Fusion Using the Brantigan i/f Cage for Posterior Lumbar Interbody Fusion and the Variable Pedicle Screw Placement System: Two-Year Results from a Food and Drug Administration Investigational Device Exemption Clinical Trial. Spine.

[33] Kurtz S.M., Devine J.N. (2007). Peek Biomaterials in Trauma, Orthopedic, and Spinal Implants. Biomaterials.

[34] Laratta J.L., Vivace B.J., López-Peña M., Guzón F.M., Gonzalez-Cantalpeidra A., Jorge-Mora A., Villar-Liste R.M., Pino-Lopez L., Lukyanchuk A., Taghizadeh E.A. (2022). 3d-Printed Titanium Cages Without Bone Graft Outperform Peek Cages with Autograft in an Animal Model. Spine J..

[35] Macki M., Hamilton T., Haddad Y.W., Chang V. (2021). Expandable Cage Technology-Transforaminal, Anterior, and Lateral Lumbar Interbody Fusion. Oper. Neurosurg. (Hagerstown).

[36] Kim J.W., Park H.C., Yoon S.H., Oh S.H., Roh S.W., Rim D.C., Kim T.S. (2007). A Multi-center Clinical Study of Posterior Lumbar Interbody Fusion with the Expandable Stand-Alone Cage (Tyche(r) Cage) for Degenerative Lumbar Spinal Disorders. J. Korean Neurosurg. Soc..

[37] Jitpakdee K., Sommer F., Gouveia E., Mykolajtchuk C., Boadi B., Berger J., Hussain I., Härtl R. (2024). Expandable Cages That Expand Both Height and Lordosis Provide Improved Immediate Effect on Sagittal Alignment and Short-Term Clinical Outcomes Following Minimally Invasive Transforaminal Lumbar Interbody Fusion (Mis tlif). J. Spine Surg..

[38] Buckland A.J., Proctor D.J. (2023). Minimally Invasive Transforaminal Lumbar Interbody Fusion with Expandable Cages. JBJS Essent. Surg. Tech..

[39] Park D.Y., Heo D.H. (2023). The Use of Dual Direction Expandable Titanium Cage with Biportal Endoscopic Transforaminal Lumbar Interbody Fusion: A Technical Consideration with Preliminary Results. Neurospine.

[40] Kucharzyk D.W., Budimir D., Waldorff E.I., Shum L.C., Vannabouathong C. (2023). The Effect of Expandable Versus Static Lordotic Interbody Implants in Minimally Invasive Spine Surgery: Patient Reported Outcomes, Sagittal Alignment, and Restoration of Disc Height and Foraminal Height. J. Spine Surg..

[41] Patel D.V., Yoo J.S., Karmarkar S.S., Lamoutte E.H., Singh K. (2019). Interbody Options in Lumbar Fusion. J. Spine Surg..

[42] Sable Product Page. https://www.globusmedical.com/expandabletechnology/sable/.

[43] Tlx 20° Procedural Animation. YouTube. https://www.youtube.com/watch?v=IDLovL6s9g0.

[44] Altera Product Page. https://www.globusmedical.com/expandabletechnology/altera/.

[45] Cannestra A.F., Peterson M.D., Parker S.R., Roush T.F., Bundy J.V., Turner A.W. (2016). Mis Expandable Interbody Spacers: A Literature Review and Biomechanical Comparison of an Expandable Mis Tlif with Conventional Tlif and Alif. Spine.

[46] Flxfit15 Articulating Expandable Interbody. https://corelinksurgical.com/product/flxfit15/.

[47] Weinstein M.A., Ayala G.A., Roura R., Christmas K.N., Warren D.H., Simon P. (2023). Transforaminal Lumbar Interbody Fusion with an Expandable Interbody Device: Two-Year Clinical and Radiographic Outcomes. N. Am. Spine Soc. J..

[48] Dualx. https://amplifysurgical.com/technology/dualx/.

[49] Coe J.D., Zucherman J.F., Kucharzyk D.W., Poelstra K.A., Miller L.E., Kunwar S. (2016). Multiexpandable Cage for Minimally Invasive Posterior Lumbar Interbody Fusion. Med. Devices Evid. Res..

[50] Lewandrowski K.U., Ferrara L., Cheng B. (2020). Expandable Interbody Fusion Cages: An Editorial on the Surgeon’s Perspective on Recent Technological Advances and Their Biomechanical Implications. Int. J. Spine Surg..

[51] Elgafy H., Behrens K. (2023). Comparing Expandable and Static Interbody Cages in Lumbar Interbody Fusion. J. Spine Surg..

[52] Cheng B.C., Swink I., Yusufbekov R., Birgelen M., Ferrara L., Coric D. (2020). Current Concepts of Contemporary Expandable Lumbar Interbody Fusion Cage Designs, part 1: An Editorial on Their Biomechanical Characteristics. Int. J. Spine Surg..

[53] Chen C., Li Q., Wang W., Ji C., Kang Y., Wang C., Zhang H., Zhang M., Zhou H., Feng H. (2022). Comparison of the Efficacy of Expandable Interbody Fusion Cage (Exp-ifc) and Non-expandable Interbody Fusion Cage (Ne-ifc) in Mis-Tlif for Lumbar Degenerative Diseases: A Systematic Retrospective Study on 62 Patients. Front. Surg..

[54] Burkus J.K. (2002). Intervertebral Fixation: Clinical Results with Anterior Cages. Orthop. Clin. North Am..

[55] Sekiguchi I., Takeda N., Ishida N. (2022). Indirect Decompression of the Central Lumbar Spinal Canal by Means of Simultaneous Bilateral Transforaminal Lumbar Interbody Fusion for Severe Degenerative Lumbar Canal Stenosis with 3 Years Minimum Follow-Up. Interdiscip. Neurosurg..

[56] Diebo B.G., Balmaceno-Criss M., Lafage R., McDonald C.L., Alsoof D., Halayqeh S., DiSilvestro K.J., Kuris E.O., Lafage V., Daniels A.H. (2024). Sagittal Alignment in the Degenerative Lumbar Spine: Surgical Planning. J. Bone Joint Surg. Am..

[57] Louie P.K., Iyer S., Khanna K., Harada G.K., Khalid A., Gupta M., Burton D., Shaffrey C., Lafage R., Lafage V. (2022). Revision Strategies for Harrington Rod Instrumentation: Radiographic Outcomes and Complications. Global Spine J..

[58] Hawasli A.H., Khalifeh J.M., Chatrath A., Yarbrough C.K., Ray W.Z. (2017). Minimally Invasive Transforaminal Lumbar Interbody Fusion with Expandable Versus Static Interbody Devices: Radiographic Assessment of Sagittal Segmental and Pelvic Parameters. Neurosurg. Focus.

[59] Vaishnav A.S., Saville P., McAnany S., Kirnaz S., Wipplinger C., Navarro-Ramirez R., Hartl R., Yang J., Gang C.H., Qureshi S.A. (2020). Retrospective Review of Immediate Restoration of Lordosis in Single-Level Minimally Invasive Transforaminal Lumbar Interbody Fusion: A Comparison of Static and Expandable Interbody Cages. Oper. Neurosurg. (Hagerstown).

[60] Stickley C., Philipp T., Wang E., Zhong J., Balouch E., O’Malley N., Leon C., Maglaras C., Manning J., Varlotta C. (2021). Expandable Cages Increase the Risk of Intraoperative Subsidence but Do Not Improve Perioperative Outcomes in Single Level Transforaminal Lumbar Interbody Fusion. Spine J..

[61] Yee T.J., Joseph J.R., Terman S.W., Park P. (2017). Expandable vs. Static Cages in Transforaminal Lumbar Interbody Fusion: Radiographic Comparison of Segmental and Lumbar Sagittal Angles. Neurosurgery.

[62] Alvi M.A., Kurian S.J., Wahood W., Goyal A., Elder B.D., Bydon M. (2019). Assessing the Difference in Clinical and Radiologic Outcomes Between Expandable Cage and Nonexpandable Cage Among Patients Undergoing Minimally Invasive Transforaminal Interbody Fusion: A Systematic Review and Meta-analysis. World Neurosurg..

[63] Stein I.C., Than K.D., Chen K.S., Wang A.C., Park P. (2015). Failure of a Polyether-Ether-Ketone Expandable Interbody Cage Following Transforaminal Lumbar Interbody Fusion. Eur. Spine J..

[64] Kim P.D., Baron E.M., Levesque M. (2012). Extrusion of Expandable Stacked Interbody Device for Lumbar Fusion: Case Report of a Complication. Spine.

[65] Chang C.C., Chou D., Pennicooke B., Rivera J., Tan L.A., Berven S., Mummaneni P.V. (2020). Long-Term Radiographic Outcomes of Expandable Versus Static Cages in Transforaminal Lumbar Interbody Fusion. J. Neurosurg. Spine.

[66] Pu X., Wang D., Gu S. (2023). Advances in Hounsfield Units Value for Predicting Cage Subsidence on Spinal Interbody Fusion Surgery. Eur. Spine J..

[67] Kim D.Y., Kwon O.H., Park J.Y. (2022). Comparison Between 3-Dimensional-Printed Titanium and Polyetheretherketone Cages: 1-Year Outcome After Minimally Invasive Transforaminal Interbody Fusion. Neurospine.

[68] Deng Z., Zou Q., Wang L., Wang L., Xiu P., Feng G., Song Y., Yang X. (2023). Comparison Between Three-Dimensional Printed Titanium and Peek Cages for Cervical and Lumbar Interbody Fusion: A Prospective Controlled Trial. Orthop. Surg..

[69] Luca A., Giorgino R. (2023). Augmented and Virtual Reality in Spine Surgery. J. Orthop..

[70] Garg D., Dubey N., Goel P., Ramoliya D., Ganatra A., Kotecha K. (2023). Improvisation in Spinal Surgery Using Ar (Augmented Reality), Mr (Mixed Reality), and Vr (Virtual Reality). Eng. Proc..

